# Efficacy of lytic bacteriophages isolated from sewage-treatment plants in Mbarara district, Uganda against ciprofloxacin-resistant *Salmonella* Typhi in a mice infection model

**DOI:** 10.1186/s12879-026-13248-z

**Published:** 2026-04-15

**Authors:** Phoebe N. Bindyo, Ntulume Ibrahim, Jackim Nabona, Godswill J. Udom, Jesca L. Nakavuma

**Affiliations:** 1 Department of Pharmacology and Toxicology, School of Pharmacy, Kampala International University, Western Campus, Ishaka-Bushenyi, Uganda; 2 Department of Biosecurity, Ecosystems and veterinary Public Health, School of Bio-Security, Biotechnical and Laboratory Sciences, COVAB, Makerere University, Kampala, Uganda; 3 Department of Microbiology and Immunology, Kampala International University, Western Campus, Ishaka-Bushenyi, Uganda; 4 Department of Pharmacology and Toxicology, Faculty of Pharmacy, Federal University, Oye-Ekiti state, Nigeria; 5 The Alliance for Phage Research and Development Uganda Ltd., Kamapala (Phage Team Uganda), Uganda

**Keywords:** Bacteriophage therapy, Multi-drug resistant species, Lytic bacteriophages, Sewage, Ciprofloxacin-resistant *S*. Typhi

## Abstract

**Background:**

Resistance to antibiotics often leads to therapeutic failure and mortality, especially in resource-limited countries including Uganda. This necessitates the search for efficacious and safe alternatives. The study determined the in vivo efficacy of isolated lytic bacteriophages from sewage treatment plants, against ciprofloxacin-resistant *Salmonella* Typhi in Mbarara district, Uganda.

**Methods:**

A laboratory based experimental study was conducted. Twelve samples obtained from two sewage treatment plants within Mbarara district, Southwestern Uganda; Kakoba and Taso were used for isolation of bacteriophages using the double agar overlay plaque assay. The isolated phages were assayed for both biological and physico-chemical characteristics. A mice infection model that included; bacteria + phage, bacteria + SM buffer, bacteria + ciprofloxacin antibiotic groups and phage only were used to assess the in vivo efficacy of bacteriophage cocktail against ciprofloxacin resistant *Salmonella* Typhi after intraperitoneal administration. The bacterial loads and phage titres from the lungs, intestines, liver, and kidney were determined using surface spreading and double agar overlay plaque assay. Data was analyzed using SPSS-26 to compute descriptive statistics and statistical significance was considered at *P* ≤ 0.05.

**Results:**

The selected phages exhibited lytic activity against ciprofloxacin-resistant *S.* Typhi, with an average host range limited to *S.* Typhi species. The phage cocktail rapidly decreased *S.* Typhi counts in blood, liver, spleen intestines, and kidney to 0 CFU/mL within 48 h compared to ciprofloxacin-treated mice. The phages were completely cleared rapidly in the spleen, kidney, and blood within 60, 96, and 120 h, respectively. Treatment with a phage cocktail caused significant reductions in the weights of the liver and intestines compared to ciprofloxacin-treated mice.

**Conclusions:**

The results highlight the lytic properties of bacteriophages against ciprofloxacin-resistant *S.* Typhi, suggesting its use in antibacterial therapy.

**Recommendations:**

The study recommends molecular characterization, pharmacokinetic, histological effects, and immunological responses to establish the safety of phage cocktails for therapeutic use in humans.

**Clinical trial number:**

Not applicable.

## Background

The emergence of fluoroquinolone (ciprofloxacin and ofloxacin)-resistant *S. enterica serovar* Typhi has led to a global burden of typhoid fever with an estimated increase in morbidity and mortality rates in developing countries including Uganda. Veeraraghavan et al. [1] reported the occurrence of multi-drug resistance caused by ciprofloxacin-resistant *S.* Typhi strains. Currently, the World Health Organization (WHO) classified fluoroquinolone-resistant Salmonella species as pathogens of high priority that necessitate research and development of new alternatives [2].

Bacteriophages are viruses that infect and kill bacterial cells. Undoubtedly, phages are the most abundant species in nature, numbering above 1 × 10^31^ [3]. On recognising and infecting their respective host cells, phages typically undergo two life cycles namely: lytic/virulent and lysogenic/temperate [4]. They are a broad group of non-living biological organisms with DNA or RNA within a protein capsid. They are naturally occurring bacterial parasites unable to reproduce independently, and thus rely entirely on a bacterial host for their survival [5]. Phages tend to attach to particular receptors on the surface of bacterial cells. They then inject their genetic material into the host cell. Afterwards, they either incorporate this material into the bacterial genome, known as “temperate” phages, and reproduce from one generation to the next, or they take control of the bacterial replication process to generate the next set of phage offspring and cause the cell to burst, known as “lytic” phages. Once a sufficient number of phage offspring is reached, varying from a few to over 1000 virus particles depending on the surrounding conditions, the lytic proteins become active and break down the peptidoglycan cell wall. This releases new phage particles, allowing the lytic cycle to start again [5].

In the early 20th century, lytic phages were utilised for the treatment of dysenteric diarrhoea [6]. In the fight against antimicrobial resistance (AMR), lytic bacteriophages are currently receiving considerable attention as antibiotic substitutes [7]. This practice is commonly referred to as “*phage therapy*” [8]. The use of naturally occurring phages for treating bacterial illness has been debated in Western medicine [5]. Nevertheless, the evolving field of phage-based antimicrobials has made significant progress beyond conventional approaches. For instance, emerging technologies, such as bioengineered chimaeras of phage-derived lytic proteins, have the potential to be considered as a new class of antibacterial agents [9].

The war between antibiotics and microbial resistance is far from being won, particularly due to the emergence of resistance even to newer antimicrobial agents [10]. This further worsens the global, regional and national burden of infections caused by microorganisms, especially the multi-drug resistant strains [11]. Thus, antimicrobial resistance is a persistent public health challenge impacting human, animal, and environmental health and world economies. This necessitates a holistic approach toward its successful management. Strategies commonly employed to manage antibiotic resistance include (i) the use of forgotten natural substances and their transformation, (ii) searching for genes specifying the biosynthesis of antibiotics, (iii) developing new vaccines against resistant bacterial strains, and (iv) looking into new antibiotic targets in pathogenic bacteria are some possible approaches [12]. The quest for novel molecules capable of inhibiting harmful microorganisms resistant to currently available antibiotics receives special emphasis [13].

Henceforth, there is a continued resistance to available antibiotics such as azithromycin and third-generation cephalosporins used in the management of *S.* Typhi infection [14]. This has left a narrow or no treatment options which puts the health sector in jeopardy [11]. As a result of this threat, urgent priority for development of novel antimicrobials such as phages is necessary [15]. Therefore, this study was designed to determine the efficacy of isolated lytic bacteriophages from sewage treatment plants in Mbarara, Uganda against ciprofloxacin-resistant *S.* Typhi in Swiss albino female mice.

## Methods

### Geographical area and study site

**Fig. 1:**
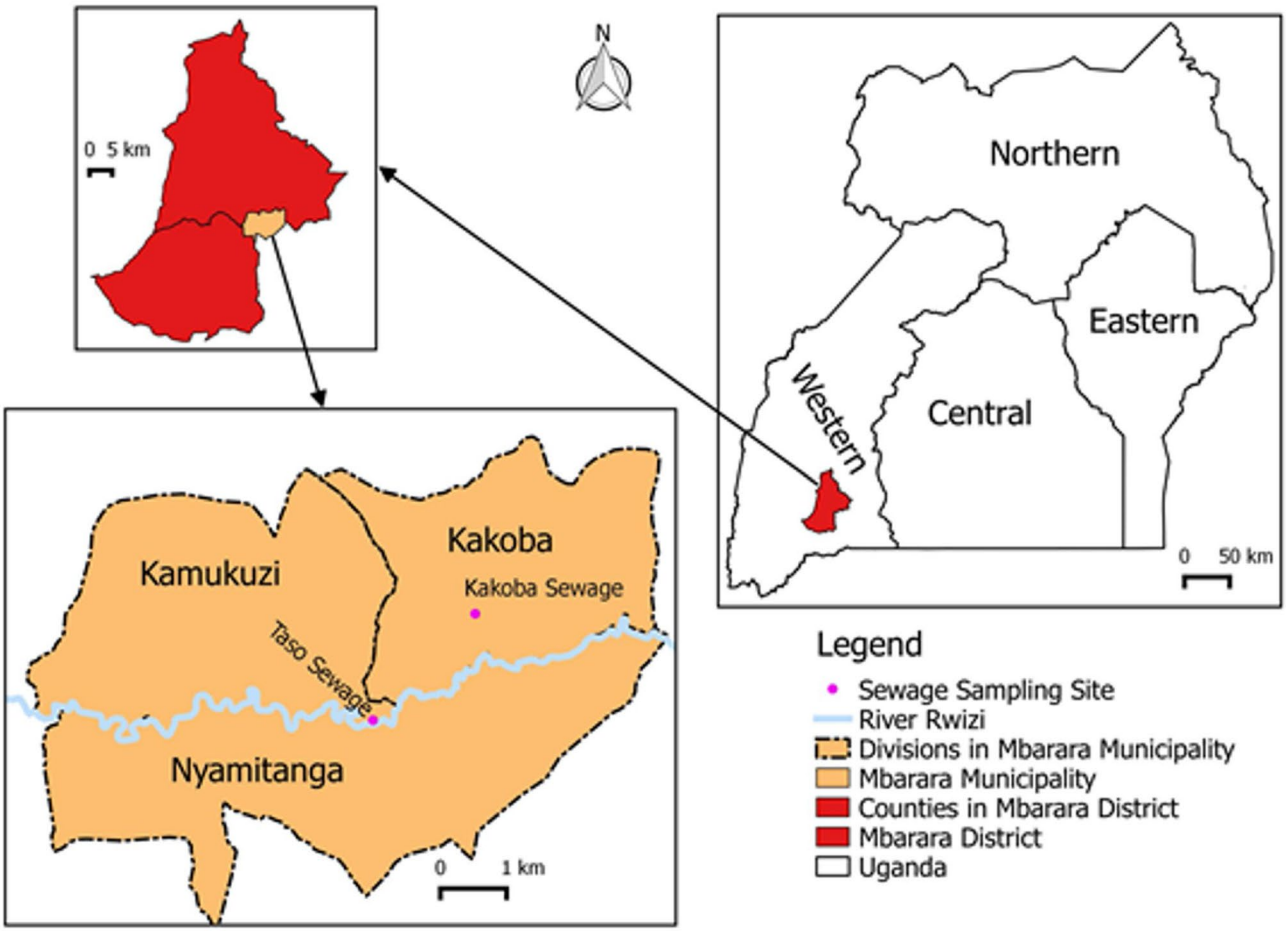
; Map of Uganda showing the sewage sampling sites

We previously collected 100 mL of each sewage samples from two sewage treatment plants located at Kakoba (0.6061˚S, 30.6741˚E) and Taso (0.6176˚S, 30.6592˚E), Mbarara municipality (00˚36’S 30˚36’E) in Mbarara district, Southwestern Uganda (Fig. 1); then isolated and characterised bacteriophages as previously described Sun et al. [16]. All study experiments were conducted in a bio-safety level 2 facility at the Institute of Biomedical Research laboratory, Kampala International University, Western Campus (KIU-WC), Ishaka-Bushenyi, Uganda.

### Test bacteria

The bacterial isolates included; *Salmonella enterica* Serovar Typhi ATCC 14,028 S strain, ciprofloxacin resistant *Salmonella enterica* Serovar Typhi isolates, whose antimicrobial susceptibility pattern were known, were acquired from the department of Microbiology and Immunology laboratory at Kampala International University, Western Campus. The bacteria were sub-cultured on xylose lysine deoxycholate agar (XLD) agar (HiMedia laboratories Pvt. Ltd, Mumbai, India) then followed by biochemical tests to confirm each bacterial species [17].

### Isolation of lytic bacteriophages

The lytic phages were isolated from sewage using the methods previously published by Sun [16] with a few minor adjustments. The sewage samples were centrifuged at 10,000 x g for 10 min at 4°C, and the supernatant was filtered using a syringe filter of 0.45 μm millipore syringe filters (Fischer Scientific Ottawa, ON). An equal volume of 20 mL of double-strength Tryptone soya broth (TSB) supplemented with 10 mM calcium chloride (CaCl_2_ 2H2O) and 5 mL of log-phase broth culture of the host bacteria (ATCC 14028 S strains of *Salmonella enterica Serovar* Typhi) at an OD600 of 0.4–0.7 was added. The enrichment mixture was incubated overnight at 37°C with 100 rpm mechanical shaking. This was followed by centrifugation at 5400 x g for 15 min at 4°C, later syringe filtered (0.22 μm millipore) the supernatant to obtain a bacterial free filtrate. Spot assay was performed using 10 µL of each serially diluted filtrate to detect the presence lytic phages based on the formation of clear discrete plaques. A control plate spotted with TSB only was added.

### Phage purification, propagation, and titre determination

Phage purification was done through selection of plaques based on morphology (clarity, turbid, haloe formation) and size [18]. The selected plaques were harvested out of the soft agar using the tip end of a sterile 1 mL micropipette tip, and then independently placed in 100 µL of phage buffer for 2 h at 4°C to allow diffusion of phages out of soft agar plug [19]. A 10-fold serial dilution was then done in sterile buffer prior to double agar overlay assay. At least five rounds of successive plaque purifications were done till uniform morphologies of plaques were consistently obtained [20].

The phages were amplified to obtain a phage stock using double agar overlay assays using a protocol adapted from Skaradzinska [20] and Kropinski et al. [21]. Plaque forming units (PFU) per milliliter of each phage was calculated using the formula below; [22].

$$\eqalign{{\rm{PFU/ml = }} & {\rm{Number}}\,{\rm{of}}\,{\rm{plaque}}\,{\rm{scounted}} \times \cr& {{\rm{1}} \over {{\rm{dilution}}\,{\rm{factor}}}} \times \cr& {{\rm{1}} \over {{\rm{Volume}}\,{\rm{of}}\,{\rm{phage}}\,{\rm{lysate}}\,{\rm{dilute}}({\rm{mL}})}} \cr} $$

The simplified polyethylene glycol (PEG) precipitation procedure [23] was used to concentrate the amplified phages. Briefly, 20 mL of phage lysate was mixed with 1 g/mL of pancreatic digest, DNase I, and RNase, then incubated at 25°C for 30 min. The lysate solution was incubated with 1 M, 1.16 g of sodium chloride on ice for 60 min. The supernatant was spun at 8300xg for 10 min at 4°C, followed with addition of 1 g of PEG 8000 at a 10% w/v working concentration. The phage particles were then precipitated by incubating the PEG/phage solution at 4°C for 60 min. The phages precipitates recovered after centrifugation at 8300xg for 10 min at 4°C were resuspended in SM buffer to produce pellets. The phage lysate suspension was cultured for one hour at room temperature. The nucleases and PEG were removed by adding an equal amount of 1% w/v chloroform to the suspension and vortexing it for 30 s. Concentrated lytic bacteriophages present in the aqueous phase were obtained after centrifugation of mixture at 4300 xg for 15 min at 4°C. The phage concentrate underwent four more rounds of Cesium Chloride (CsCl)-gradient ultracentrifugation for further purification before being kept in 50% glycerol at -80°C and in TSB at 4°C [24].

### Host range

The host range of the isolated phages was ascertained using a spot assay technique [25]. One hundred micro litre of log-phase broth culture of ciprofloxacin resistant *Salmonella enterica* Serovar Typhi, *Klebseilla pneumonie*,* Proteus mirabilis*,* Shigella spp*,* Escherichia coli*, and *Staphylococcus aureus* at OD_600_ of 0.4–0.7 were mixed with 3 mL of molten TSA (0.7% agar, w/v) kept at 45°C and after, carefully poured on pre-prepared TSA, swirled and then allowed to solidify at room temperature. This was followed by spotting 10 µL of each serially diluted phage lysate on the soft agar containing bacterial lawns. The spotted lysates were allowed to dry at room temperature for approximately 15 min, and then later plates were incubated for 24 h at 37°C. Formation of clear inhibition zone and/plaques at the spotted area was indicative of lytic activity of the test bacteria. Control plates consisted of only a lawn of test bacteria. Three independent experiments were performed.

### Determination of biological and physico-chemical characterization of lytic phage isolates

#### Optimal multiplicity of infection

The broth culture of the test bacteria at log-phase (OD_600_ of 0.4) in single strength TSB supplemented with 10mM Calcium chloride was added into various eppendorf tubes containing phage lysates at various ratios of 10^− 5^, 10^− 4^, 10^− 3^, 10^− 2^, 10^− 1^, 1, 10^1^, 10^2^, 10^3^, 10^4^ pfu cfu^− 1^. The mixture was incubated for 2 h at 37°C. After incubation, the mixture was centrifuged at 9000 g for 3 min to obtain a supernatant which was filtered through a 0.22 μm syringe filter. The phage titres in the filtrates were determined using double agar overlay method in three independent experiments. The optimal MOI used for the subsequent experiments was one with the highest phage titer within 2 h of incubation [24].

#### Adsorption time

The time required for the phages to adsorb onto the bacterial host cell wall was determined according to Mirzaei and Nilsson [18]. Five hundred micro litre of the overnight bacterial culture was added to 25 mL of TSB, incubated with shaking at 100 rpm until OD_600_ reached 0.4 (~ 3 h, 10^8^ CFU/mL). Then 900 µL of the culture was aliquoted into 16 tubes labeled 0 s, 30 s, 1, 5, 10, 15, 30 and 60 min in duplicate. To each of these tubes, 100 µL of the phage at titer of 10^9^ PFU/mL was added at MOI of 1 followed by incubation at 37°C. At every time interval, samples were kept on ice briefly, then centrifuged at 21,000 g for 5 min and then filtered supernatants through 0.22 μm millipore syringe filters (Fischer Scientific Ottawa, ON). The titres of the phages were determined using the double-agar layer method on the 10-fold serially diluted aliquots. The experiment was done in triplicates.

#### One-step growth curve

The study utilized the methods described by Yazdi et al. [26]. with slight adjustments to determine the latent time and phage burst size. In brief, overnight host bacterial cells at OD_600 nm_ 0.4 was pelleted by centrifugation at 5400xg for 5 min and then re-suspended in TSB supplemented with 10 mM CaCl_2_ 2H_2_O (2 ml) (~ 10^9^ CFU/ml). The phage lysates at multiplicity of infection (MOI) of 0.01 were added to the bacterial suspension and then let to be adsorbed for the predetermined adsorption time (5–15 min) at 37°C, then centrifuged at 13,000×g for 5 min. This was followed by re-suspending the bacterial pellets in 20 mL of TSB supplemented with 10 mM CaCl_2_ 2H_2_O and then incubated for 120 min at 37°C. At 10 min interval, 100 µL of the sample was collected and assayed for phage titres using the double-agar layer method. The assay was performed in three independent experiments.

#### Efficiency of plating (EOP) analysis

The phages with broader host range were selected for EOP determination using the double agar overlay plaque assays as documented by [18]. This was computed as a comparison of the lysis efficiency of phages on the target hosts with their isolation hosts. Briefly, 10-fold serial dilutions of phage lysate suspension were mixed with 1 mL of host and target host bacterium and then incubated for 10 min at 37°C before being plated as double agar layers. Later, the plates were incubated at 37°C overnight for plaque formation to calculate the phage titres formed. The experiment was performed in triplicates. The EOP values of the selected phages were calculated as a ratio of the phage titers produced on the target bacterium to the phage titers produced on their original isolation hosts. The assays were performed in triplicates to obtain average number of titres per bacteria tested. The EOP value for each phage-bacterium combination was determined as high production effectiveness (ratio of 0.5), medium production effectiveness (0.1 to 0.5), poor production (EOP 0.001–0.1), and inefficient production (0.001) [18].

#### Thermal and pH stability

The stability of the isolated bacteriophages at various temperature and pH conditions was determined according to methods described by Qamar [22]. The thermal stability of phages was assessed by pre-incubation at various temperatures; 25°C, 30°C, 40°C, 50°C, 60°C, 70°C, 80°C, and 90°C in a water bath for 2 h. For pH stability assessment, 100 µL of phage lysate at 1.0 × 10^9^ PFU/ml was added in 1 mL of phosphate buffered saline (PBS) at pH of 1, 2, 4, 6, 7, 8, 10, 12 contained in different eppendorf tubes. Sodium hydroxide or hydrochloric acid was used in adjusting the various pH ranges. The mixture was incubated at 37 °C for 2 h. Aliquots were used to ascertain phage titers at time intervals of 10 min using the double-agar overlay method. All experiments were done in triplicates.

#### Phage stability during storage

The stability of bacteriophage at various reserve temperatures was periodically determined storing the phage lysates at 4°C, -20°C, -40°C, and −80°C: 0, 10, 20, and 30 days. The experiments were done when phage lysates were both mixed with glycerol at concentration of 10%, 15%, 20%, 25%, 30%, and 50% and without glycerol. After every storage interval, remaining phage titres were assayed employing the double-agar layer method [18].

#### Preparation of phage cocktails

The four phages included in the cocktail were selected based on their burst size, in vitro inhibitory efficacy, high productive efficiency of plating (EOP), wide host range against the target bacteria and highly stable at various pH and temperature ranges. The selected phages were combined in equal proportions at a 10^6^ PFU/mL concentration.

#### In vitro bacterial inhibitory efficacy of phage cocktail

The in vitro therapeutic efficacy of phage cocktail was conducted using an ELISA to monitor the rate of bacterial growth when treated with different concentrations of phage cocktails as earlier applied by Köhne et al., [27] with slight modification. In 5 mL of TSB, a fresh overnight culture of the test bacteria was prepared at 37°C for 18 h. The culture was then pelleted down at 4000 g centrifugation for 5 minutes and washed twice with fresh TSB containing 10 mM CaCl_2_ 2H_2_O. Two hundred microliters of bacterial culture adjusted to 1 × 10^5^ CFU/mL was poured into each well of a 96 well microtire plate and then followed with addition of prepared cocktail at MOIs of 1, 10, and 100. The plates were incubated at 37°C for 24 h and optical densities of each reading were recorded after every 30 min using Ao Absorbance Microplate Reader (Azure biosystems, Dublin, CA, USA). Control wells with only test bacteria were included and experiment was done in triplicates.

#### Experimental animals

Thirty-two female Swiss albino mice aged 6 to 8 weeks were gotten from the Kampala International University animal house and kept in sterilised polypropylene cages with sterile paddy husk as bedding at the Animal Unit, Department of Pharmacology and Toxicology, School of Pharmacy, KIU-WC. The animals were quarantined and allowed to acclimatise to laboratory conditions for 7 days before the experiment. During this time, they were allowed access to food and water *ad libitum*. The animals were handled in tandem with the ARRIVE guidelines. Experimental animals within the same weight range were caged together and marked with picric acid as either the head, back, tail, plain, or right ear to facilitate simple identification during drug administration. This was done to minimise potential differences that may arise during the experiment. The six cages utilised were assigned numbers based on their locations on the rack. Ethical approval (BSU-REC-2023-139) was obtained from the Research Ethics Committee, Bishop Stuart University under the Uganda National Council for Science and Technology.

#### In vivo therapeutic efficacy of phage cocktails

As previously described [28–30], in vivo therapeutic efficacy of phage cocktails against ciprofloxacin-resistant *S. enterica serovar* Typhi was done in a mice infection model.

#### Preparation of bacteria suspension for injection

Ultimately, those purified and confirmed isolates of ciprofloxacin-resistant *S. enterica serovar* Typhi were grown to an early exponential growth phase (OD_600nm_ 0.75) in TSB, centrifuged at 10,000 x g to obtain pellets and washed twice in sterile injection-grade saline. The bacterial pellets were then resuspended in 100 mL of sterile injection saline, and enumerated [30].

#### Mice bacteraemia model

Thirty-two mice were randomly allotted to four groups (*n* = 10 each) and treated as shown in Table 1. The experimental animals in groups 2–4 were intraperitoneally administered 200 µL of ciprofloxacin-resistant *S. enterica serovar* Typhi (5 × 10^8^ CFU/mL). After that, within a 24–48 h timeframe, they were carefully and regularly observed for signs of bacterial infection, and those who met the criteria for euthanasia were promptly euthanised in line with guidelines for animal handling [30]. Confirmation of Salmonella infection in the mouse model was carried out through a culture of samples of faeces intestinal contents using selenite F broth, which served as an enrichment medium to separate Salmonella from faeces. After establishing bacterial infection, purified phage cocktails in SM buffer at determined optimal MOI (approximately 3 × 10^9^ PFU/mL) in a fixed injection volume (200 µL ≡ 0.2 mL) were administered intraperitoneally to groups 1 and 2. Mice in Groups 3 and 4 received 200 µL SMB and 100 mg/kg of ciprofloxacin intraperitoneally, respectively. The mice were euthanised under halothane anaesthesia and sacrificed at 12, 24, 36, 48, 60, 72, 84, 96, 108 and 120 h post-phage treatment (1 mouse per group at every time interval), and selected internal organs were eviscerated and blood samples were collected from each euthanised mouse.

**Table 1:** Presence of lytic phages in analyzed samples

Sewage treatment plant	Location of sample site	Sample IDs	Presence of phages
Kakoba sewage treatment plant	Kakoba division, Mbarara district	KK001	+
		KK002	+
		KK003	-
		KK004	+
		KK005	-
		KK006	+
		KK007	-
Taso sewage treatment plant	Kamukuzi division, Mbarara district	TA001	+
		TA002	+
		TA003	+
		TA004	+
		TA005	-
		TA006	+
		TA007	+

+; lytic phages present -; Lytic phages absent

### Determination of bacterial counts and phage titres

Firstly, 0.5 mL of whole blood was collected by puncturing the orbital plexus of each test mouse into a tube containing 20 µL of heparin. Selected internal organs (bilateral lungs, intestines, liver, spleen, and kidney) were collected and aseptically weighed. Then, 1 g of each organ sample was homogenized in 1 mL sterile phosphate-buffered saline (PBS) using a tissue homogenizer with a rotor-stator tissue. The viable counts of *S. enterica serovar* Typhi were determined by the surface spreading method using 100 µL of 10-fold serially diluted sample from each organ and heparinized whole blood on XLD agar. The phage titres from each of the above samples were identified using the double agar overlay plaque assays [31]. The emergence of phage resistance was evidenced by bacterial loads that remained statistically unchanged across the sampled time intervals.

### Data analysis

Statistical analysis was done using SPSS version 17 (SPSS Inc., USA), and statistical significance between the groups was analysed via one-way analysis of variance (ANOVA) followed by Duncan’s test as a post-hoc test. The results were presented as tables, graphs and pictures. The statistical significance was established at *p* < 0.05. To minimise bias, the statistician was blinded to the treatment regimen employed in this study.

## Results

### Presence of lytic bacteriophages in analyzed sample filtrates

The double agar overlay plaque was used to detect the presence of lytic bacteriophages in the enriched sewage samples on bacterial lawns. The results showed that four and six bacterial free filtrates from both Kakoba and Taso sewage treatment plants respectively harbored lytic phages against the isolation host *S*. Typhi (Table 1).

### Plaque morphology and titres of phage lysates

**Fig. 2:**
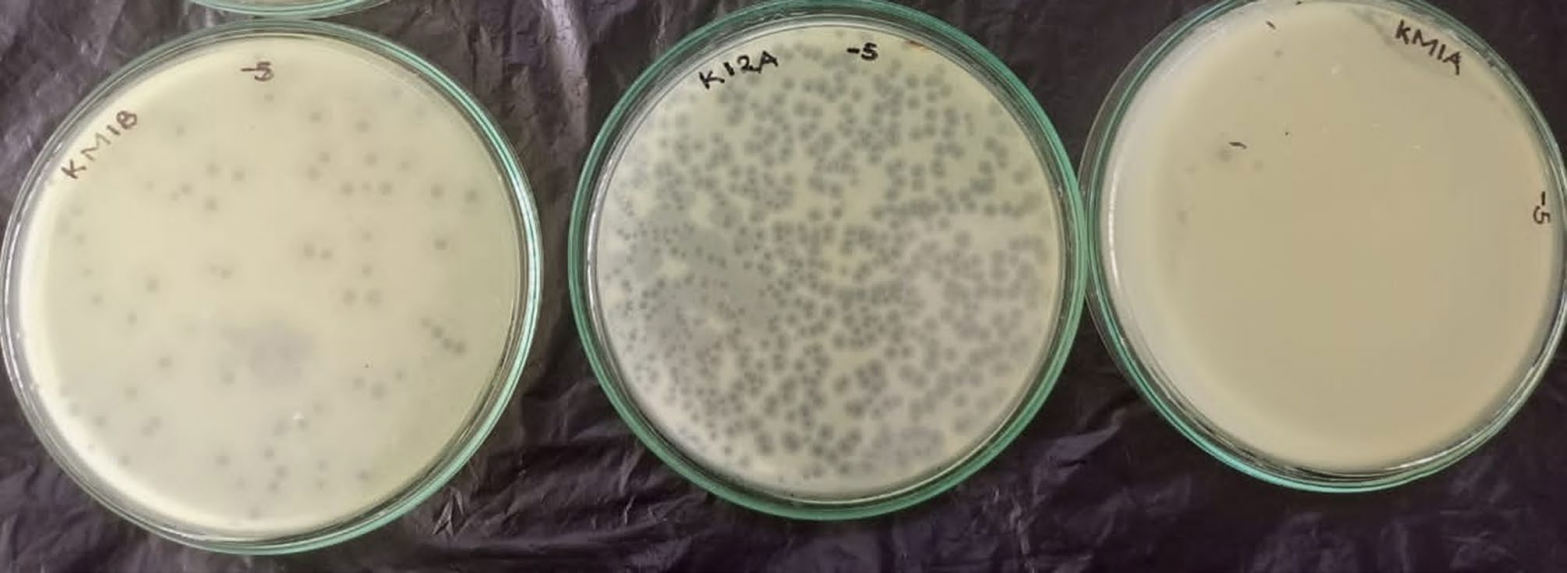
Double agar overlay plaque assay for isolation of lytic phages. Plates labeled KM1B, KI2A exhibit discrete plaques and no plaques on plate labeled KM1A

The study isolated 10 lytic phages against *S*. Typhi of which only four phages exhibited virulent plaque morphologies and wide host ranges based on the obtained percentages of cross infectivity and these included; KK001, TA001, TA003, TA06. The phages produced clears plaques ranging from 0.2 mm to 1.0 mm on the host bacteria. The selected phages had high initial titres that ranged from 2 × 10^17^ PFU/mL for phage TA001 to 19 × 10^12^ PFU/mL for phage TA003 (Table 2; Fig. 2).

**Table 2:** Plaque morphologies of isolated phages

Phage ID	Plaque morphology			
	Plaque size (mm)	Characteristics	Initial phage titres (PFU/mL)	
KK001	0.5	Pinpoint, sharp clear	2 × 10^15^	
TA001	0.2	Pin point, sharp clear	2 × 10^17^	
TA003	1.0	Pinpoint-small, sharp clear	19 × 10^12^	
TA006	0.4	small, sharp clear	16 × 10^14^	

### Host range of isolated bacteriophages

**Fig. 3:**
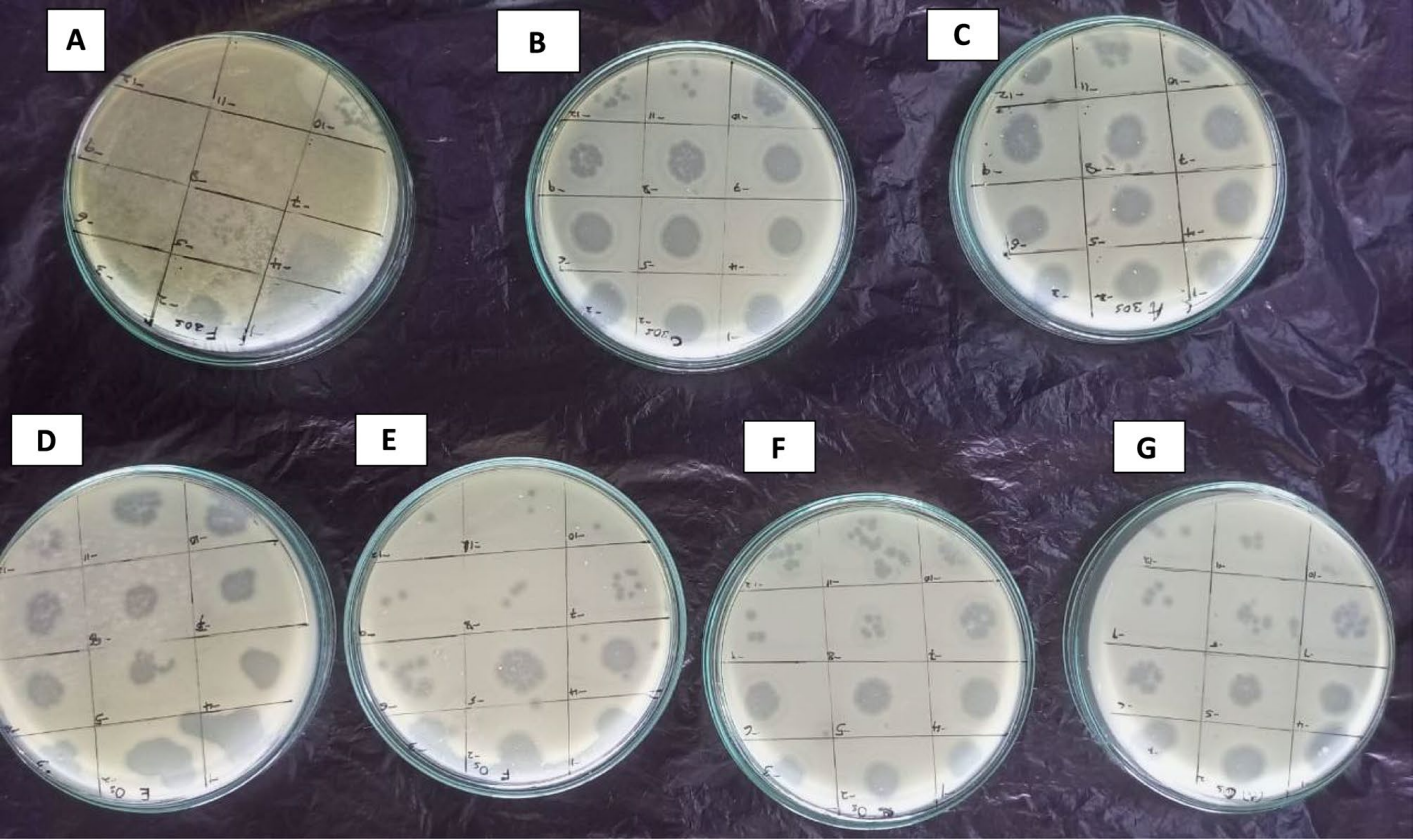
Host range of isolated phages using spot assay. Zones of inhibition showed on **Plates A**; Shgella species, **B**; S. Typhi ATCC 6539, **C**; S. Typhi cipro R−HN−01, **D**; *S*. Typhi cipro R−CD−01, **E**; S. Typhi cipro R−HN−02, **F**; S. Typhi cipro R−HN−03, **G**; S. Typhi cipro R−HN−04

The isolated bacteriophages exhibited an average host range with percentage cross infectivity that ranged from 1(8.3%) to 6(50.0%) specifically against the species of *S.* Typhi. Phages TA001 exhibited better host range of 50% with a spectrum activity against Shigella species as compared to other phages that had specificity to only against only Salmonella genus (Table 3; Fig. 3). Phages KK001, TA001, TA003 and TA006 were considered to have broader spectrum of activity and had an average EOP of 0.35, hence were selected for further characterization and in vivo efficacy studies.

**Table 3:**
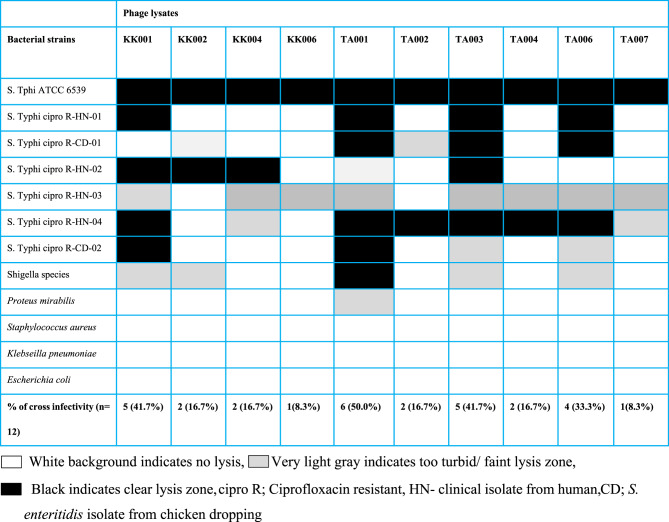
Host range of isolated lytic phages

### Adsorption time of phages

**Fig. 4:**
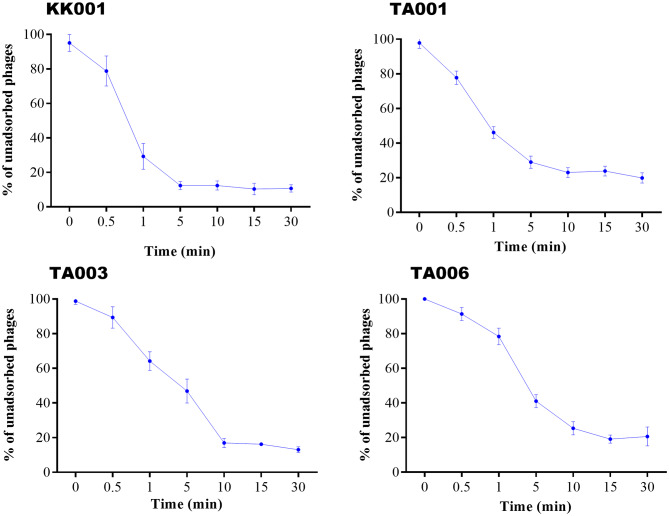
Percentage adsorption time of phages

The selected four lytic bacteriophages against *S. Typhi* exhibited adsorption time of 5 min, 10 min, 10 min and 15 min for phages KK001, TA001, TA003, and TA006 respectively as shown in Fig. 4.

### One step growth curve of phages

**Fig. 5:**
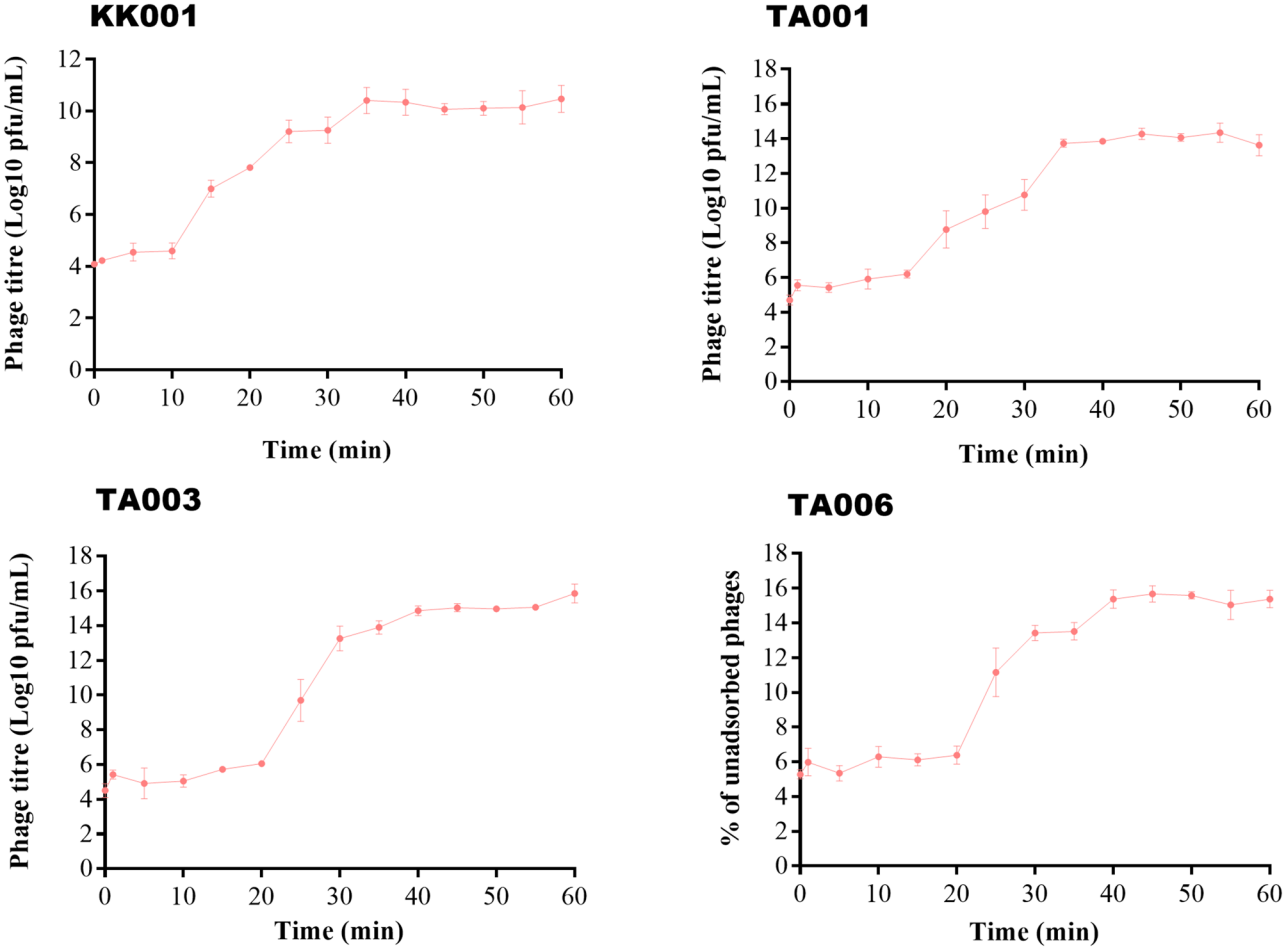
Adsorption time of phages

The phages had latent periods of 10 min for phage KK001 and 20 min for phages TA001, TA002, and TA006. Furthermore, the phages exhibited high burst sizes that ranged from 163 PFU/mL, PFU/mL, 183 PFU/mL, 255 PFU/mL and 286 PFU/mL for phage TA001, KK001, TA006 and TA003 respectively. All phages had a burst period of 20 min as shown in Fig. 5.

### Thermal and pH stability of phages

**Fig. 6:**
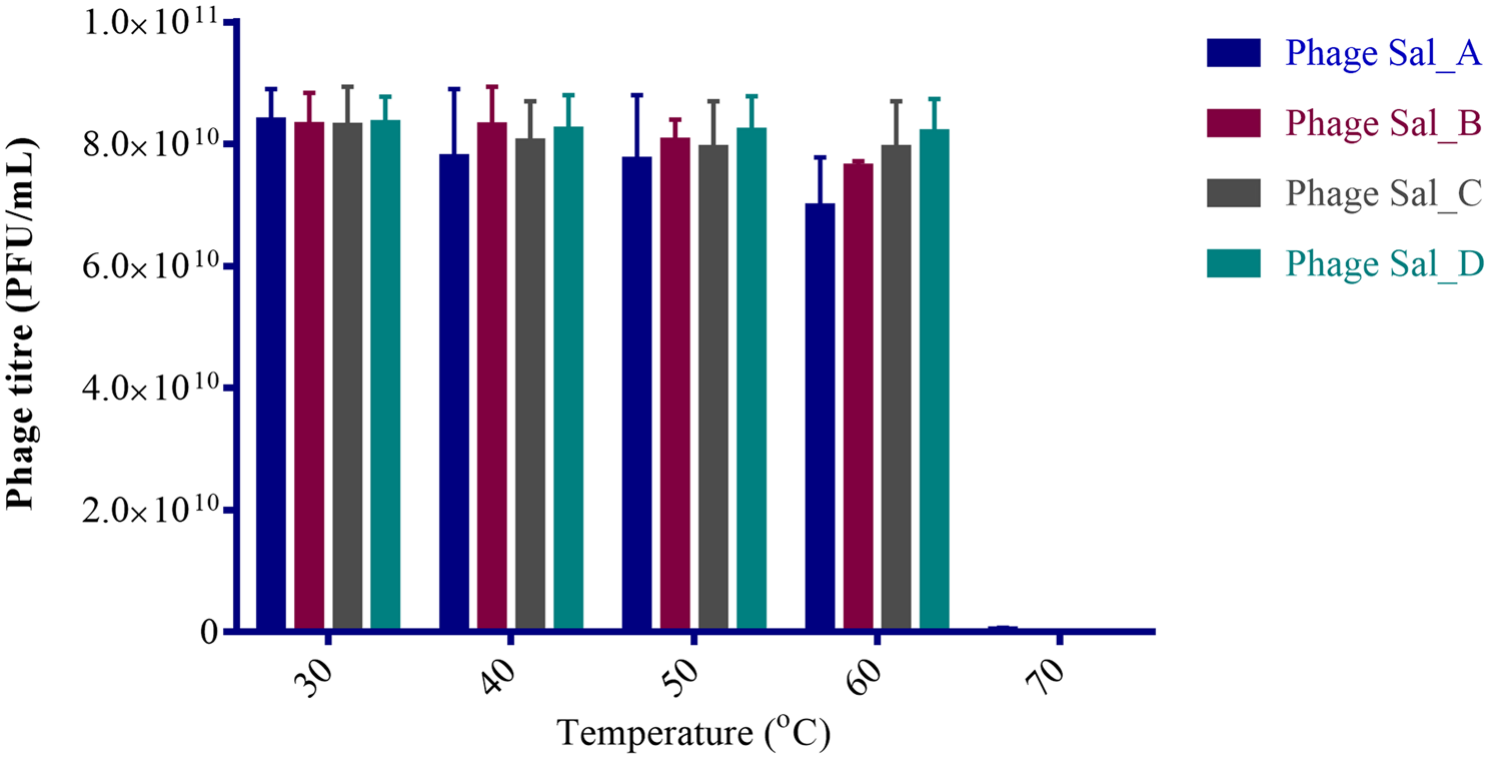
Thermal stability of selected phages

**Fig. 7:**
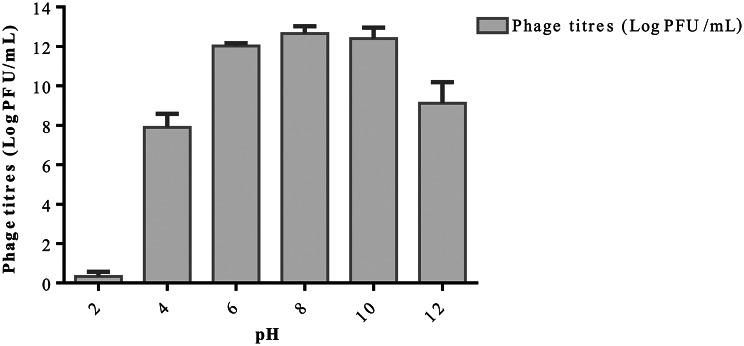
pH stability of selected phages

All phages exhibited stability across different temperatures between 30 and 60°C. However, the phages were denatured at temperatures of 70°C (Fig. 6). The phages exhibited significant decrease in phage titres at pH (*p* = 0.0003) and pH 12 (*p* = 0.00065) while at pH2, phages were rapidly denatured to 0.33 log PFU/ml as shown in Fig. 7.

### In vivo therapeutic efficacy of phage cocktails

The cocktail exhibited highest inhibitory efficacy against *S*. Typhi within 4 h and then followed by a slightly bacteria growth significantly lower than the control. There was slight resurgence of phage mutants after 18 h.

### In vivo antibacterial effects of phage cocktail on *S.* Typhi counts in infected mice models

**Fig. 8:**
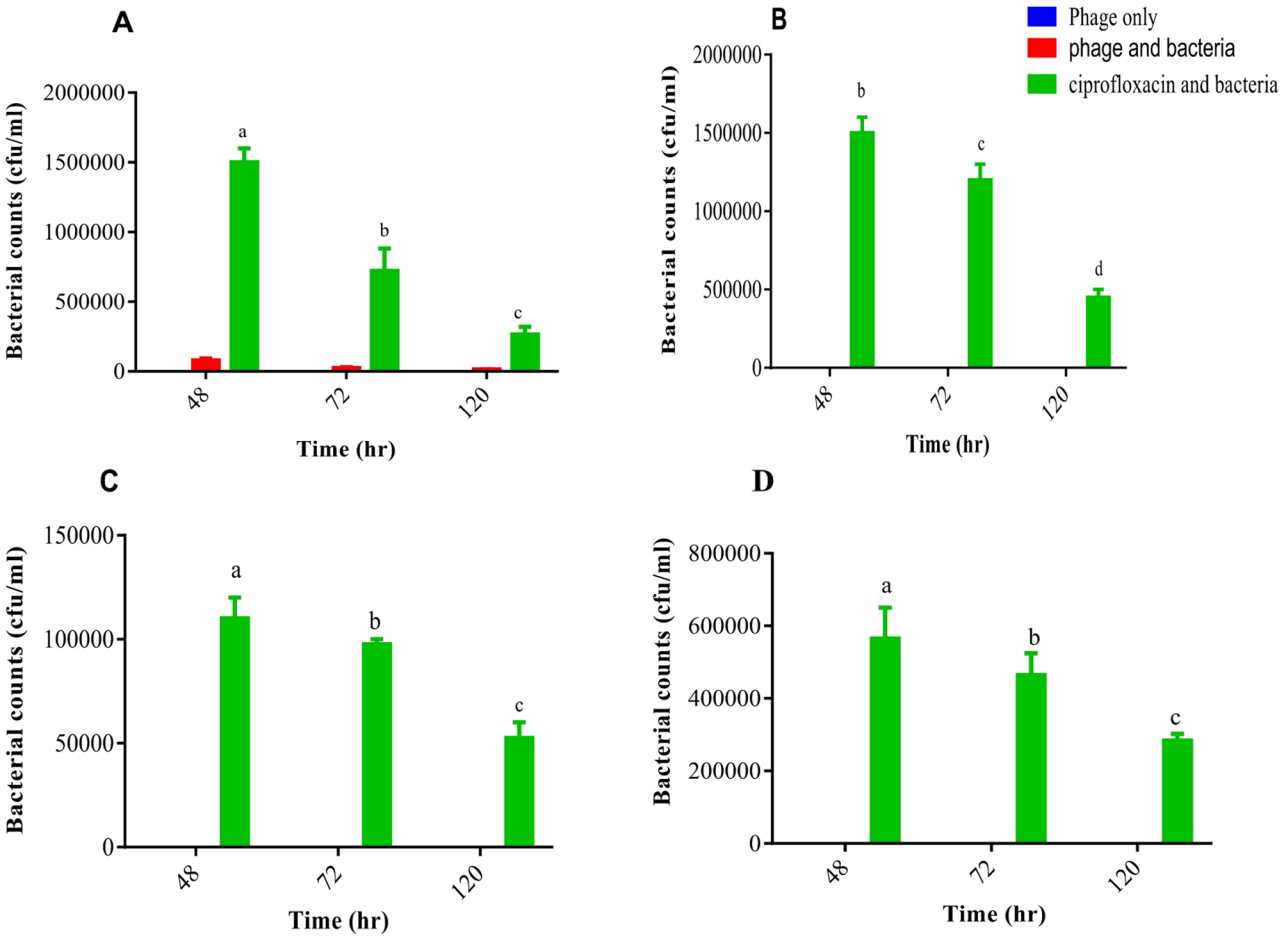
In vivo antibacterial effect of phage cocktail on bacterial loads in different organs of S. Typhi infected mice model. **A**: Intestines; **B**: Liver; **C**: blood; **D**: Kidney. ^a, b, c^: statistical significance between phage and ciprofloxacin-treated groups at 48, 72, and 120 h, respectively

There was a gradual/rapid decrease in the bacteria loads for the phage-treated group till 120 h compared to the ciprofloxacin-treated group. The phage cocktail rapidly decreased the *S.* Typhi loads in the blood, liver and kidneys to 0 CFU/mL within 48 h compared to the group that received ciprofloxacin as showed in Table 4. The intestinal bacterial loads of *S.* Typhi were significantly reduced between 12 h and 48 h. However, between 48 and 120 h, the loads showed no significant difference (*p* = 0.9943), suggesting the emergence of phage-resistant mutants (Fig. 8).

**Table 4:** ; allocation of treatment groups

S/*N*	Group (*n* = 8)	Doses	Duration
1	Group 1 (Phage only)	PhaC (200 µL)	48 h
2	Group 2 (Bacteria + phage)	PhaC (200 µL)	“
3	Group 3 (Bacteria + SMB)	SMB (200 µL)	“
4	Group 4 (Bacteria + Cipro)	Cipro (100 mg/kg)	“

SM = Salt of magnesium and sodium buffer; PhaC = phage cocktail; Cipro = ciprofloxacin

### Bacteriophage titres in selected mice organs

**Fig. 9:**
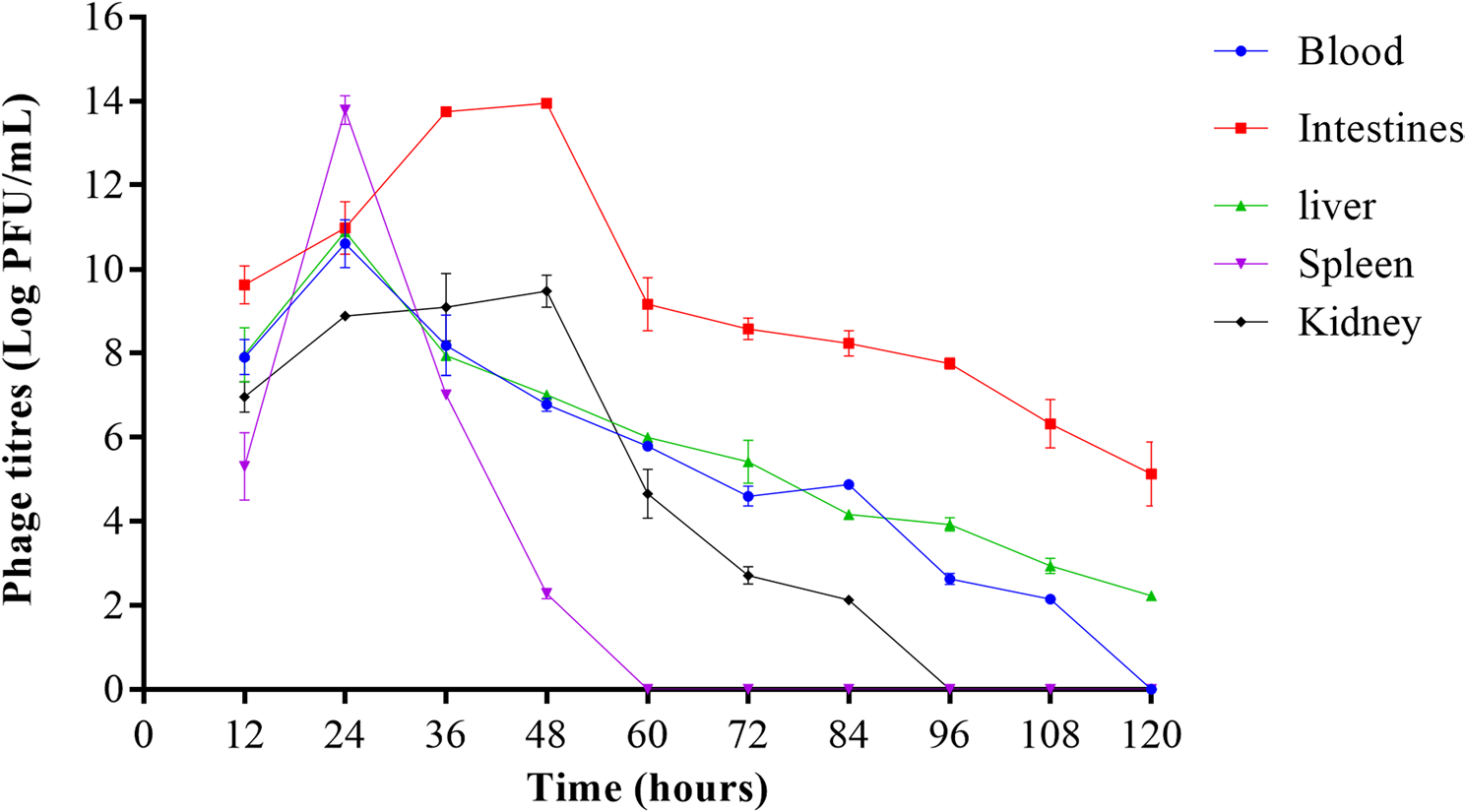
Trend of phage titres in different organs of *S.* Typhi infected mice model over time

In the bacteriophage-treated groups, the titres of bacteriophages were significantly elevated in all organs 12 h post phage administration. However, within hours, the titres gradually increased in the intestines to 13.9 ± 0.01 log PFU/mL compared to the blood and liver. After 120 h, titres in both the liver and blood significantly decreased to 0 and 2.28 ± 0.02 log PFU/mL compared to the intestines, where titres decreased exponentially to 9.16 ± 0.63 after 60 h; then gradually to 5.12 ± 0.77 log PFU/mL after 120 h (Fig. 9).

At 12 h, the spleen had significantly lower phage titres than the blood, intestines, liver and kidneys. However, this was rapidly increased at 24 h; and then quickly and completely cleared at 60 h. There was no statistical difference in the phage titres in the blood and liver between 12 and 108 h. However, phage titres in the blood were eliminated compared to those of the liver. For the kidneys, there was an initial gradual increase in the bacterial loads at 48 h; then followed by a rapid decrease to 0 CFU/mL at 96 h. This significantly differed from the intestines, liver, and blood titres. The phages were rapidly and completely cleared in the spleen, kidneys, and blood within 60, 96, and 120 h, respectively (Fig. 9).

### Effect of bacteriophages on the weights of selected organs of *S.* Typhi infected mice model

At 48 h, there was no significant difference (*p* > 0.05) in the weights of kidneys and liver in the treatment group (Fig. 9). However, there was a significant decrease in the weights of intestines and liver for the phage-treated group compared to the ciprofloxacin-treated group and the untreated infected group.

**Fig. 10:**
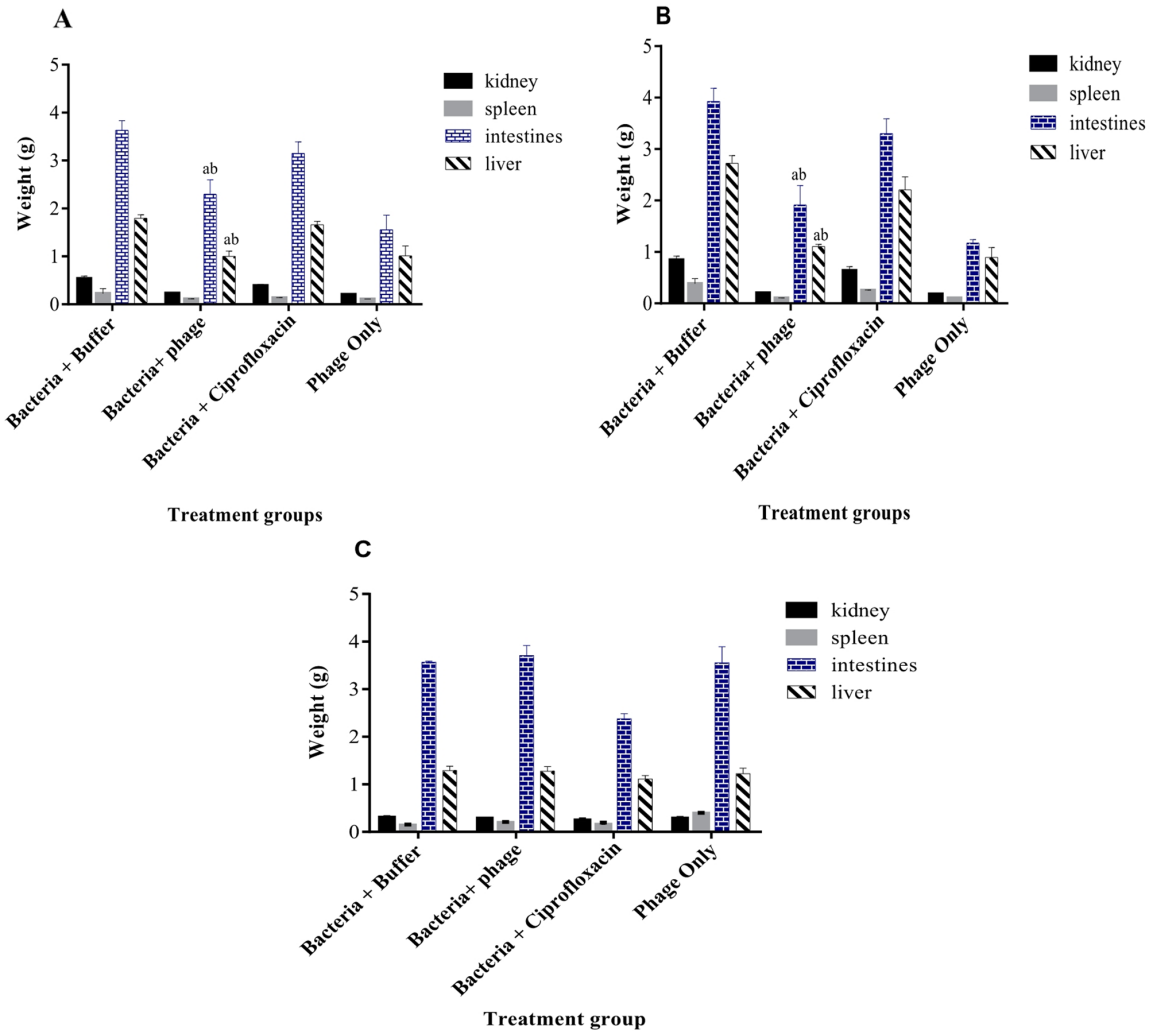
; Effect of bacteriophages on weights of *S.* Typhi infected mice model- **A**: 48 h; **B**: 72 h; **C**: 120 h. ^a^ statistical significance between bacteria + buffer and bacteria + phage; ^b^ statistical significance between bacteria + phage and bacteria + ciprofloxacin

At 72 h post phage treatment, there was no significant difference in the kidney and liver weights within the treatment group. Similarly, the ciprofloxacin-treated group had no significant reduction in intestine and liver weights compared to the untreated group (i.e., Group 3). However, there were significant reductions in the weights of the intestine and liver compared to the untreated group. Also recorded were significant reductions in the liver and intestine of mice in the phage-treated group compared to the ciprofloxacin-treated group (Fig. 10).

At 120 h post-treatment of infected mice with phage, the weights of kidneys significantly decreased compared to the untreated group. In contrast, that of the ciprofloxacin-treated group had no significant difference compared to the untreated group. The spleen weights were significantly decreased in the phage-treated group compared to the untreated group. Similarly, the weights of intestines were significantly reduced in the phage-treated group compared to the untreated and ciprofloxacin-treated groups. However, there was no significant difference in intestine weights in the ciprofloxacin-treated mice compared to the untreated group (*p* = 0.3436). Additionally, the weight of the liver in the phage-treated group was significantly (*p* < 0.0001) lower compared to the untreated and ciprofloxacin-treated groups; while that of the ciprofloxacin-treated group had no significant difference compared to the untreated group (*p* = 0.9163). Notably, throughout the entire experiment, the weights of the liver, intestines, kidneys and spleen of mice in the phage-treated group were not significantly different from those administered only phage (i.e., Group 1).

## Discussion

The emergency of Fluoroquinolone (ciprofloxacin and ofloxacin) resistant *Salmonella enterica* subsp. *enterica* serovar Typhi has led to an increased burden of typhoid fever in developing countries like Uganda. Currently, the World Health Organization classified fluoroquinolone-resistant Salmonella as a pathogen of high priority that necessitates research and development of new alternatives such as Phage therapy [32].

The present study isolated four virulent phages that formed clear plaques on a wide range of test bacteria which clearly indicated the presence of lytic phages in sewage effluents against the *S.* Typhi [27]. Similarly, Wang et al. [33]. and Bao et al. [28]. reported lytic phages against *Salmonella enterica* subsp. *enterica* serovars. The broad host range exhibited by the four selected phages make them more desirable over a cocktail in terms of cost effective treatment of typhoid fever [34].

The isolated phages exhibited diverse plaque morphologies that could be attributed by both intrinsic factors of the phage and extrinsic factors such as; temperature, agar density, and incubation time [35]. Yazdi et al. [26] reported significant rates of phage isolation and variation in plaques morphologies when enrichment media is supplemented with divalent cations which ultimately affects the adsorption rate. According to Gallet et al. [36]., formation of small plaques could be attributed to slowed diffusivity of phages with larger morphological structures, then shortens the diffusion distance which ultimately reduces the number of nearby lysed cells.

The phages showed high degree of thermal and pH stability making them suitable for therapeutic use [37–39]. The moderate thermal stability has been linked to the presence of cross linking disulfide bonds between coat protein dimers / phage icosahedral capsid [40, 41]. Montso et al. [42]., and Sharma et al. [37] reported significant reduction in phage titres at temperatures beyond 50°C. Still, our findings were contrary to those of Wang et al. [43] who reported Salmonella phages that survived at temperatures of 4°C to 70°C.

The in vitro efficacy exhibited by the cocktail showed synergic potential of the individual phages and slowed resurgence of phage resistant mutants as previously reported in related studies [35]. The exhibited trade-off and fitness cost-loss of major phage receptors could ultimately make the resistant strains once again susceptible to ciprofloxacin antibiotics [44].

Our study showed that the phage cocktail rapidly increased after the intraperitoneal administration and cleared the bacterial (*S.* Typhi) loads in the blood, intestines, liver and kidneys to 0 CFU/mL within 48 h compared to the ciprofloxacin-treated group. This underscores the fact that bacteriophages stay wherever there are bacteria. This agrees with the findings of Bao et al. [28] which reported 80% phage protection in *S. Typhi* infection mice. Though the determinants of success for phage therapy are multifactorial, phage dose greatly influences it. The majority of phages infect and kill just the bacteria that possess their specific receptor, which ultimately determines their host range [45]. Phages exhibit varying degrees of host specificity, with some being unique to certain strains, while others have the potential to infect a wide range of bacterial strains and even other genera [46, 47]. Bacteria have developed multiple strategies to prevent infection by lytic phages, and phages have equally possessed a wide range of ways to overcome this resistance. The strategy for bacterial resistance can involve changes or downregulation and/or loss of receptors and incorporation of phage DNA into the “clustered regularly interspaced palindromic repeats/CRISPR associated system (CRISPR/Cas) system” [48]. To counteract this mechanism of bacterial resistance, phages identify new or modified receptors and the anti-CRISPR genes [49]. The predominant lytic phages linked to human disease-causing organisms and the gut microbiota belong to the Caudovirales and Microviridae orders. Caudovirales, sometimes known as “tailed phages,” have double-stranded DNA genomes, whereas Microviridae are tailless viruses with single-stranded DNA [50, 51].

Furthermore, Wang et al. [43]. reported similar findings of the therapeutic potential of phages against *S. enterica serovar Abortusequi* that induces abortion in mice. Therefore, our choice of a mouse model was selected as a better way to provide reliable information on the efficacy and safety of phage therapy for future studies in clinical trials [28]. The study adopted the intraperitoneal route of phage administration since it has been proven as the most successful way of rapid distribution of phages into systemic circulation. For instance, Dhungana et al. [29] reported a significantly high phage concentration (2.3 × 105 PFU/mL) after 4 h administration through the intraperitoneal route. A similar MOI of phage administration was consistently reported in related studies. Additionally, the intraperitoneal route for phage administration has been documented to successfully protect mice against S. Typhi mortality [28] and this is in tandem with the methods and findings of Wang et al. [43]. which showed protection of mice from the lethal dose of *S. enterica serovar Abortusequi*. Notably, the intraperitoneal administration of phages to healthy mice is not associated with the stimulation of pro-inflammatory cytokines (TNF-α and IL-6) [29]. The oral route could impede phage absorption in the gastrointestinal tract due to slowed permeability and gut acidity [29]. However, besides its use in the treatment of ovarian cancers, the intraperitoneal route used in the present study is mostly used in research animals and rarely in humans for treatment administration [32].

The reduction in bacterial loads observed in the present study could also be attributed to the mounted immune response that eventually eliminates the remaining/phage-resistant strains in circulation [28, 43]. Oftentimes, bacterial infections are associated with inflammation due to the release of inflammatory cytokines and other mediators. Previous studies reported that within 12 and 48 h post-administration, phages significantly reduced the levels of circulating inflammatory cytokines (e.g., IFN-1, TNF-α, IL-10, and IL-17) to approximately normal levels in the placenta and serum in a bacterial animal model [29, 43]. Thus, protecting bodily organs from the toxic effects of inflammation. This could explain the gradual reduction in the weights of organs in mice treated with phages compared to the untreated group due to continuous inflammation of the tissues.

While these results are suggestive, the specific impact of inflammation on organ weight loss requires confirmation through histopathological analysis, as recommended by this study. It should be noted that organ weight changes reported in this study are indirect measures and do not replace histopathological assessment.

Nevertheless, our study recovered phages from the blood, liver, intestines, spleen and kidneys indicating the ability of phages to survive gut pH conditions, temperatures, and osmolality and eventually traverse GIT’s barrier into the mice’s systemic circulation to reach different organs. This agrees with the findings of Dhungana et al. [29]. Though the mechanism of phage translocation in the gut remains unknown, however, some studies suggested various factors such as; phage titre, stomach acidity, interactions with gut immune cells and micropinocytosis - the process of incorporating macromolecules or other chemical compounds into cells occurs through membrane invagination and the subsequent pinching off of small vesicles [29, 52]. We reported higher phage titres in the intestines compared to other body organs, indicating that the intestines are favourable to higher replication rates due to the high bacterial loads in the host. Nonetheless, all organs had phage particles which could suggest that phages can cross the epithelial barrier and enter extraintestinal sites [28]. This correlates with the gradual reduction in bacterial loads due to the lytic activity of circulating phages. In contrast, the gradual reduction of phages in blood and other organs within 120 h reported in this study disagrees with a related study that documented rapid reduction of phages in the blood, liver, and kidneys, after 24 h and then completely cleared after 72 h [29]. Nonetheless, phage titres within the spleen were completely cleared after 60 h, a rate faster than that reported by Dhungana et al. [29]. The initial accumulation of phage titres in the liver and spleen, and the rapid clearance suggest that the reticuloendothelial system (e.g., CD8α dendritic cells and Kupffer cells) and filtering organs plays a key role in clearing phages, and this agrees with previous reports [29, 53].

## Conclusion

From our findings, the phage cocktail exhibited potential in vivo efficacy against ciprofloxacin-resistant *S. Typhi* in which it completely cleared the bacteria from the blood, liver, spleen, intestines and kidneys within 48 h compared to positive control. Data from this study lend credence to the therapeutic exploitation of bacteriophages. However, it is imperative to conduct related studies using samples from other sewage treatment plants located in different regions of Uganda to ascertain if they too harbour virulent bacteriophages against ciprofloxacin-resistant *S. Typhi* strains. Since phage therapy necessitates the use of phages that do not possess toxins or antimicrobial resistance (AMR) genes, it is crucial to genetically analyse (genotypic profiling) the virulent phages and determine their morphological structures by the utilisation of transmission electron microscopy. Additionally, it is necessary to verify the immunogenicity and pharmacokinetic profiling of these phages before their application in phage treatment. This highlights the necessity for additional research focused on bacteriophages as safe alternative antibacterial agents, particularly in light of the global, regional and national menace and/or devastating impact of antimicrobial resistance.

## Data Availability

All datasets used to generate the table and figures are available upon a reasonable request to the corresponding author.
